# Validation of prevalent diabetes risk scores based on non-invasively measured predictors in Ghanaian migrant and non-migrant populations – The RODAM study

**DOI:** 10.1016/j.puhip.2023.100453

**Published:** 2023-11-23

**Authors:** James Osei-Yeboah, Andre-Pascal Kengne, Ellis Owusu-Dabo, Matthias B. Schulze, Karlijn A.C. Meeks, Kerstin Klipstein-Grobusch, Liam Smeeth, Silver Bahendeka, Erik Beune, Eric P. Moll van Charante, Charles Agyemang

**Affiliations:** a Department of Public and Occupational Health, Amsterdam UMC, University of Amsterdam, Amsterdam Public Health Research Institute, Meibergdreef 9, Amsterdam, the Netherlands; b Non-communicable Disease Research Unit, South African Medical Research Council, Cape Town, South Africa; c Department of Global and International Health, School of Public Health, Kwame Nkrumah University of Science and Technology, Kumasi, Ghana; d Department of Molecular Epidemiology, German Institute of Human Nutrition Potsdam‐Rehbruecke, Nuthetal, Germany; e Center for Research on Genomics and Global Health, National Human Genome Research Institute, National Institutes of Health, Bethesda, MD, USA; f Julius Global Health, Julius Center for Health Sciences and Primary Care, University Medical Center Utrecht, Utrecht University, Utrecht, the Netherlands; g Division of Epidemiology and Biostatistics, School of Public Health, Faculty of Health Sciences, University of the Witwatersrand, Johannesburg, South Africa; h Department of Non‐Communicable Disease Epidemiology, London School of Hygiene and Tropical Medicine, London, UK; i MKPGMS-Uganda Martyrs University, Kampala, Uganda; j Department of General Practice, Amsterdam UMC, University of Amsterdam, Amsterdam Public health Research Institute, Meibergdreef 9, Amsterdam, the Netherlands; k German Center for Diabetes Research (DZD), Germany; l Institute of Nutritional Science, University of Potsdam, Germany

**Keywords:** Diabetes risk prediction, External validation, Sub-Saharan Africa population, Migrant population

## Abstract

**Background:**

Non-invasive diabetes risk models are a cost-effective tool in large-scale population screening to identify those who need confirmation tests, especially in resource-limited settings.

**Aims:**

This study aimed to evaluate the ability of six non-invasive risk models (Cambridge, FINDRISC, Kuwaiti, Omani, Rotterdam, and SUNSET model) to identify screen-detected diabetes (defined by HbA1c) among Ghanaian migrants and non-migrants.

**Study design:**

A multicentered cross-sectional study.

**Methods:**

This analysis included 4843 Ghanaian migrants and non-migrants from the Research on Obesity and Diabetes among African Migrants (RODAM) Study. Model performance was assessed using the area under the receiver operating characteristic curves (AUC), Hosmer-Lemeshow statistics, and calibration plots.

**Results:**

All six models had acceptable discrimination (0.70 ≤ AUC <0.80) for screen-detected diabetes in the overall/combined population. Model performance did not significantly differ except for the Cambridge model, which outperformed Rotterdam and Omani models. Calibration was poor, with a consistent trend toward risk overestimation for screen-detected diabetes, but this was substantially attenuated by recalibration through adjustment of the original model intercept.

**Conclusion:**

Though acceptable discrimination was observed, the original models were poorly calibrated among populations of African ancestry. Recalibration of these models among populations of African ancestry is needed before use.

## 1 Introduction

There is a compelling argument for frequent screening among people who are at high risk of getting diabetes [1]. Early-stage detection would enable appropriate interventions that can slow the progression of diabetes, and limit related complications, disability, and mortality [2]. This has led to a booming field in clinical research where mathematical models, also known as prediction models, prediction equations, or risk scores, used to assess the probability of an individual having (diagnostic prediction scores) or developing (prognostic prediction scores) diabetes [3]. Depending on the scope of predictors included, diabetes risk scores may be distinguished as invasive and non-invasive risk scores. Invasive risk scores include laboratory-acquired biomarkers as predictors while non-invasive risk scores do not [4,5].

Due to differences in genetic factors, environment, and body composition, the varying performance of several risk models has been reported when evaluated in different settings [[6], [7], [8], [9]]. Risk scores are evaluated on their discriminative power to distinguish people with the outcome of interest from those without [10], and their calibration property, which is the model's ability to accurately estimate the probability of the event under consideration [11]. Diagnosis of diabetes by invasive biochemical laboratory testing has been found to perform better than noninvasive models [9]. However, due to the need for large-scale screening in high-risk populations especially in resource-poor settings, the use of noninvasive risk models is more cost-effective than invasive risk models [5,11].

Available diabetes risk scores mostly originate from non-African populations and have rarely been validated in people of African ancestry [12,13]. The few validation studies conducted have reported varying predictive performance for different noninvasive diabetes models in sub-Saharan African populations [9,14]. It is therefore not clear which diabetes risk score may be most suitable for diagnostic prediction of diabetes among people of African ancestry both within Africa and in the diaspora. This work is therefore aimed at evaluating the predictive performance of six noninvasive diabetes risk scores (Cambridge, FINDRISC Original, Kuwaiti, Omani, Rotterdam, and SUNSET model) to detect new cases of diabetes and prediabetes among Ghanaian migrants and non-migrants.

## 2 Methods

### 2.1 Study population and design of the study

Data from the Research on Obesity and Diabetes among African Migrants (the RODAM study) was used as the basis for validation of models. The detailed rationale and design of the RODAM study are published elsewhere [15]. The current analysis includes 4843 participants with an average age of 45.5 years and between 25 and 70 years, who have no previous diagnosis of diabetes and were not on treatment for diabetes (Figure A1 Appendix).

### 2.2 Ethical clearance

Ethical clearance was obtained from relevant ethics committees at each of the study sites: Ghana (Kwame Nkrumah University of Science & Technology: CHRPE/AP/200/12), Netherlands (Amsterdam University Medical Center: W12-062#12.17.0086), Germany (Charité University Berlin: EA1/307/12) and UK (London School of Hygiene & Tropical Medicine: 6208). The aim of the study and the use of participant data for the purposes of understanding obesity and diabetes related outcomes was explained to each participant before written consent was taken. Participant confidentiality was assured during data collection, storage and analysis.

### 2.3 Model selection

Diabetes prediction models included in this study were prevalence models with non-invasive predictors that have been previously validated in sub-Saharan African ancestry populations [14], with predictor variables available in the RODAM study database. Six non-invasive prevalent diabetes models were selected for this study. These include the Cambridge Risk Score [16], FINDRISC Original [1], Kuwaiti diabetes risk score [17], Omani diabetes risk score [18], Rotterdam Predictive Model [19], and the SUNSET Risk Score [20].

Details of the method employed including, criteria for inclusion in the current study, physical measurement, used outcome variables and cut-offs, statistical methods for predictive performance measurement and recalibration are place in the supplementary sheet due to manuscript word limits.

## 3 Results

### 3.1 Participants’ characteristics and prevalent screen-detected diabetes

Of the 4843 Ghanaian participants selected for this analysis, 57.2% were residents in Europe and 42.8% were residents in Ghana. The prevalence of screen-detected diabetes was 4.6% in the overall population, with an additional 30% classified as prediabetes. A higher prevalence of screen-detected diabetes was found among participants resident in Europe with 5.5% screen-detected diabetes compared to 3.4% of those in Ghana (Table 1).

**Table 1:** Socio-demographic characteristics and diabetes status of RODAM Study participants.

Parameter	Total	Europe	Ghana
Total population	4843(100)	2768(57.2)	2075(42.8)
**Age (yrs)**^a^	45.5 ± 11.7	45.7 ± 10.4	45.7 ± 12.2
**Gender**			
Male	1823(37.6)	1160(41.9)	663(32.0)
Female	3020(62.4)	1608(58.1)	1412(68.0)
**Participant Occupation**			
Upper non-manual	393(8.1)	265(9.6)	128(6.2)
Lower non-manual	881(18.2)	481(17.4)	400(19.3)
Skilled manual	409(8.4)	126(4.6)	283(13.6)
Unskilled manual	1551(32.0)	1039(37.5)	512(24.7)
Farmer	544(11.2)	6(0.2)	538(25.9)
Unknown	1065(22.0)	851(30.7)	214(10.3)
**Body Adiposity**			
BMI (Kg/m^2^)^a^	28.0 ± 5.6	29.4 ± 5.0	24.9 ± 5.1
WC (cm)^b^	92.1(90.7-93.2)	94.4(93.5-95.1)	84.6(83.4-85.5)
Hypertension	2181(45.0)	1508(54.5)	673(32.4)
**Diabetes Status**^c^			
Normoglycemia	3170(65.5)	1473(53.2)	1697(81.8)
Prediabetes	1452(30.0)	1144(41.3)	308(14.8)
Screen-detected Diabetes	221(4.6)	151(5.5)	70(3.4)

Data are presented as figures and corresponding percentages.

a Age & BMI are presented as mean and standard deviation of the mean.

b WC is presented as median and 95% confidence interval of the median.

c Diabetes HbA1c ≥48 mmol/moL, prediabetes HbA1_C_ ≥39 and < 48mmol/moL and normoglycemia HbA1_C_ <39 mmol/mol.

### 3.2 Prediction of prevalent screen-detected diabetes

All six diabetes risk models assessed produced acceptable discrimination (AUC = 0.70 to 0.75) for the detection of screen-detected diabetes in the total study population. The Cambridge risk model showed the highest discrimination with an AUC of 0.75 (95% CI, 0.73-0.76), with the Rotterdam risk score having the lowest AUC of 0.70 (95% CI 0.69-0.72). The SUNSET risk model was the best performing model for screen-detected diabetes among Ghanaians resident in Ghana (AUC = 0.78) but the worst performing model among Ghanaians resident in Europe (AUC = 0.70) (Table 2 and Figure A2). Among the population living in Ghana, model performance did not differ between urban and rural dwellers (Table A5). As seen in Supplementary Table A6, poor discrimination was observed for all models when fasting blood glucose was used to define the outcome variable.

**Table 2:** Predictive performance of diabetes risk score for screen-detected diabetes among the population of Ghanaians living in Europe and Ghanaians living in Ghana.

Parameter	Cambridge	FINDRISC	Kuwaiti	Omani	Rotterdam	SUNSET
**All Individuals**						
**AUC**	0.75(0.73-0.76)	0.74(0.72-0.75)	0.73(0.71-0.73)	0.72(0.71-0.73)	0.70(0.69-0.72)	0.73(0.72-0.75)
**Sensitivity**	65.6%	66.1%	73.9%	59.6%	73.9%	69.7%
**Specificity**	72.3%	68.0%	60.0%	74.4%	59.5%	69.9%
**Criterion**	>0.1776	>0.054	>0.0249	>0.1638	>0.0718	>0.3726
**p value**	<0.0001	<0.0001	<0.0001	<0.0001	<0.0001	<0.0001
**Male Ghanaians**						
**AUC**	0.76(0.74-0.78)	0.75(0.73-0.77)	0.73(0.71-0.75)	0.76(0.74-0.78)	0.71(0.69-0.74)	0.77(0.75-0.78)
**Sensitivity**	76.1%	84.4%	50.0%	77.2%	75.0%	81.5%
**Specificity**	69.1%	56.3%	85.9%	65.3%	61.1%	64.9%
**Criterion**	>0.1771	>0.014	>0.1082	>0.1171	>0.0718	>0.3243
**p value**	<0.0001	<0.0001	<0.0001	<0.0001	<0.0001	<0.0001
**Female Ghanaians**						
**AUC**	0.73(0.72-0.75)	0.73(0.71-0.75)	0.73(0.71-0.74)	0.70(0.68-0.71)	0.69(0.67-0.71)	0.72(0.71-0.74)
**Sensitivity**	82.5%	66.4%	77.8%	46.8%	73.0%	73.8%
**Specificity**	53.5%	72.4%	56.3%	82.6%	58.6%	64.8%
**Criterion**	>0.0839	>0.09	>0.0249	>0.2442	>0.0691	>0.3778
**p value**	<0.0001	<0.0001	<0.0001	<0.0001	<0.0001	<0.0001
**Ghanaians Living in Ghana**						
**AUC**	0.73(0.71-0.75)	0.73(0.71-0.75)	0.72(0.70-0.74)	0.71(0.69-0.73)	0.64(0.62-0.66)	0.78(0.76-0.79)
**Sensitivity**	74.3%	62.0%	67.1%	72.9%	61.4%	64.3%
**Specificity**	66.6%	73.9%	69.5%	63.4%	67.3%	80.0%
**Criterion**	>0.0839	0.0465	>0.0249	>0.1171	>0.0718	>0.3778
**p value**	<0.0001	<0.0001	<0.0001	<0.0001	<0.0001	<0.0001
**Ghanaians Living in Europe**						
**AUC**	0.74(0.72-0.76)	0.71(0.69-0.72)	0.72(0.70-0.74)	0.72(0.70-0.73)	0.72(0.71-0.74)	0.70(0.68-0.72)
**Sensitivity**	64.2%	57.5%	62.8%	67.6%	62.8%	72.3%
**Specificity**	73.5%	75.0%	73.5%	67.0%	71.1%	62.8%
**Criterion**	>0.2621	>0.096	>0.1081	>0.1638	>0.0824	>0.3726
**p value**	<0.0001	<0.0001	<0.0001	<0.0001	<0.0001	<0.0001

Data are presented as AUC (95% CI), AUC- Area under the curve. A score of 0.50 indicates no discrimination; 0.50<AUC<0.70 poor discrimination; 0.70≤AUC<0.80 acceptable discrimination; 0.80≤AUC<0.90 excellent discrimination; 0.90≤AUC outstanding discrimination; and a score of 1.00 perfect discrimination.

### 3.3 Prediction of prevalent prediabetes

All the assessed diabetes risk equations had a modest to acceptable discrimination power for the detection of prediabetes, with the Rotterdam risk model showing the lowest AUC 0.64 (0.63-0.66) and the Cambridge risk model the highest 0.70 (0.69-0.71) in the overall sample (Table A3 and Figure A5, Appendix).

### 3.4 Comparison of model performance

The Rotterdam risk model was found to have been outperformed by all other risk scores assessed among Ghanaians living in Ghana. The FINDRISC and Cambridge diabetes risk models outperformed the Rotterdam diabetes risk model in the prediction of screen-detected diabetes irrespective of gender. The SUNSET exhibited superior discriminative power over the Omani and the Kuwaiti among Ghanaian residents in Ghana (Table 3).

**Table 3:** Differences in AUC among the six diabetes risk scores in the prediction of screen-detected diabetes among Ghanaian living in Europe and Ghana.

Parameters	Cambridge	FINDRISC	Kuwaiti	Omani	Rotterdam	SUNSET
**All Individuals**						
**AUC**	**0.743**	**0.732**	**0.726**	**0.720**	**0.703**	**0.729**
**FINDRISC**	0.0103					
**Kuwaiti**	0.0179	0.0076				
**Omani**	0.025*	0.0146	0.0070			
**Rotterdam**	0.0421***	0.0318	0.0242	0.0171		
**SUNSET**	0.0112	0.00084	0.0068	0.00138	0.0309	
**Male Ghanaians**						
**AUC**	**0.762**	**0.766**	**0.734**	**0.763**	**0.714**	**0.765**
**FINDRISC**	0.0042					
**Kuwaiti**	0.0279	0.0321				
**Omani**	0.0012	0.0030	0.0291			
**Rotterdam**	0.0476*	0.0519*	0.0197	0.0488*		
**SUNSET**	0.0028	0.0015	0.0307	0.0016	0.0504	
**Female Ghanaians**						
**AUC**	**0.731**	**0.741**	**0.727**	**0.698**	**0.691**	**0.722**
**FINDRISC**	0.0101					
**Kuwaiti**	0.0040	0.0141				
**Omani**	0.0332	0.0433*	0.0292			
**Rotterdam**	0.0405**	0.0506**	0.0365*	0.0073		
**SUNSET**	0.0092	0.0193	0.0052	0.0240	0.0313	
**Ghanaians Living in Ghana**						
**AUC**	**0.734**	**0.760**	**0.722**	**0.711**	**0.643**	**0.777**
**FINDRISC**	0.0263					
**Kuwaiti**	0.0111	0.0373				
**Omani**	0.0228	0.0491	0.0117			
**Rotterdam**	0.0909***	0.117**	0.0798*	0.0681*		
**SUNSET**	0.0431	0.0168	0.0542*	0.0659**	0.134***	
**Ghanaians Living in Europe**						
**AUC**	**0.740**	**0.708**	**0.721**	**0.717**	**0.722**	**0.698**
**FINDRISC**	0.0327*					
**Kuwaiti**	0.0188	0.0139				
**Omani**	0.0234	0.0092	0.0046			
**Rotterdam**	0.0185	0.0142	0.0003	0.0049		
**SUNSET**	0.0424**	0.0097	0.0236	0.0189	0.0239	

Data are presented as **AUC** and difference in AUC. *p<0.05, **p<0.01, ***p< 0.001.

### 3.5 Model calibration and recalibration

In general, we observed an overestimation of the risk of diabetes by the original models with no agreement between the expected risk and the observed risk (p<0.0001 for the Hosmer & Lemeshow statistic). Intercept adjustment substantially attenuated these differences (Table 4). The Kuwaiti and Cambridge models underestimated the risk at the lower deciles and overestimated the risk at the higher deciles after intercept adjustment and the reverse was observed for the Rotterdam and the SUNSET models. The Omani models produced an undulating pattern of both underestimation and overestimation. Both the original and the recalibrated Rotterdam models produced an intercept >0. All the original models of the remaining five models produced an intercept <0. This did not change after intercept adjustment for 4 out of the 5 remaining models, except for the SUNSET model, which produced an intercept >0 after recalibration. Three models (Kuwaiti, Omani, and Cambridge) produced a slope <1. The other two models (Rotterdam and SUNSET) produced a slope >1. In general, both the original and recalibrated models exhibited low Brier scores. The exception was observed for the original SUNSET model, which had a Brier score of 0.129. Eyeballing of the calibration graphs revealed that the Omani model provided sufficient attenuation for satisfactory recalibration (Figures A3 and A4, Appendix). The deciles of risk plots are shown in Figures A6 and A7 in Appendix.

**Table 4:** Chi-Square from Hosmer and Lemeshow tests for the calibration of diabetes risk models for screen-detected diabetes before and after recalibration through intercept adjustment among Ghanaians residents in Ghana and Ghanaian Migrants.

Parameter	All	Sex		Location	
		Female	Male	Europe	Ghana
**Cambridge**					
Original	915.4	496.1	437.9	308.5	157.5
Recalibrated	156.5	26.7	32.1	17.4	28.1
**Kuwaiti**					
Original	134.7	116.1	24	97.2	44.9
Recalibrated	67.8	102.6	33.1	119.5	48.3
**Omani**					
Original	300.1	236.5	74.4	193.5	111.4
Recalibrated	33.4	30.7	26.9	25.5	21.1
**Rotterdam**					
Original	87.2	42.1	53.2	44.7	46.3
Recalibrated	34	18	31.3	29.5	13.8
**SUNSET**					
Original	1902.5	1354.8	553.8	1163.7	740.6
Recalibrated	28.6	41.6	33.1	44.6	32.5

Values are Hosmer & Lemeshow Chi-square value obtain for calibration of the original model and after intercept adjustment of the model (recalibrated model). The bigger the value, the more deviation there is between the expected prevalence predicted by the model and the observed prevalence. At a 2 degree of freedom, the p-value for the Hosmer & Lemeshow test was <0.0001 for all chi-square values.

## 4 Discussion

In general, the six selected non-invasive models assessed had acceptable discrimination for the detection of diabetes and modest-to-acceptable discrimination for prediabetes. Findings from the few validation studies of the selected diabetes models among populations of African ancestry are consistent with modest-to-acceptable discrimination [14,21]. The discrimination observed for the Cambridge model in the current study (AUC: 0.75) was higher than in two earlier reports [22,23] (AUC: 0.67, and C-statistic: 0.67 respectively). The Performance of the FINDRIC original model in the current study was found to be lower than what was earlier reported in Nigeria [24], similar to that reported in Kenya [21], but higher than that reported in Botswana [25]. With an AUC range of 0.70 to 0.73, the Kuwaiti, Omani, and, Rotterdam predictive models recorded higher discrimination ability in the RODAM study compared to those reported for the Bellville South study (AUC: 0.64-0.68) [22], but lower discrimination ability compared to those recorded among African Surinamese (AUC: 0.78-0.81) and Ghanaians (AUC: 0.74-0.76) in the HELIUS study [26]. The lower performance of these models in the current study compared to that of the HELIUS study may be explained by the inclusion of participants with known diabetes (prevalent diabetes) in the HELIUS analysis.

The Rotterdam model was outperformed by all the other models among participants living in Ghana, but it produced acceptable predictions among participants living in Europe. Masconi, Matsha [22] also reported that the Rotterdam model had the lowest validation performance range among the South African population. The SUNSET model was the best performing model for Ghanaians living in their home country, but the worst among Ghanaian migrants in Europe. Thus, the two models have exhibited inverse geographical inconsistencies among a homogeneous population. This could be explained by variations in the baseline risk profile across settings [27], such as higher obesity and hypertension rates among participants living in Europe than those living in Ghana [26]. However, such geographical variations in performance may also be attributed to the influence of migration and its impact on diabetes risk profile. Earlier findings from the RODAM study have shown the probability of developing diabetes is higher among Ghanaians living in Europe compared to rural Ghanaians with the same level of BMI [28]. A plausible explanation seems that Ghanaians in the home country may engage in more physical activity with a similar level of BMI compared to their peers living in Europe. Therefore, the interaction between captured and non-captured behavioural and biomedical risk factors in the models could account for these differences in performance [26]. Among the participants included in this study, 46.4% of Ghanaians living in Europe confirmed having tested for diabetes within the last two years to sampling as compared to 5% of testing among those in Ghana. Since the design excluded participants with known diabetes, a higher degree of testing standards in the European setting may have yielded both differences in screen-detected diabetes prevalence and inclusion rates. However, with a higher prevalence of screen-detected diabetes among Ghanaians living in Europe (5.5%) compared to those in the home country (3.4%), the assumption that a higher frequency of previous diabetes screening in the population living in Europe would affect the performance of models among Ghanaians in Europe seems implausible.

Model composition, which includes the number of variables (parsimonious/extended) and type of variables (behavioural/anthropometric) may influence the performance of the model. For example, most studies suggest a higher predictive ability of diabetes by waist circumference (WC) than body mass index (BMI) [29,30]. Thus, a model using WC or both WC and BMI may be a better predictor of diabetes than a model using BMI only. Apart from the variables common to all the models, the Cambridge includes smoking and current steroid treatment, the FINDRISC includes physical activity, fruit, and vegetable intake while the SUNSET includes ethnicity, resting heart rate, and family history of premature cardiovascular diseases. These variables may share/pick up different aspects of the underlying diabetes risk of these models and therefore have the potential of influencing the performance. However, the benefit of these additional variables in the extended models in the current study is not clear, since the difference in performance among the models with additional variables did not differ significantly from those without.

Poor calibration performance by original models and the inability of model updating/improvement through intercept adjustment, to achieve total attenuation that was seen in the current study, corroborate with earlier studies [22,26]. The initial difference in prediction by the original models could result from a true difference in the prevalence of the outcome in the development and validation populations or from a difference in the methods of outcome measurement between the development and the validation study [22,26]. The persistence of non-optimal calibration after intercept adjustment has been attributed to the sensitivity of the Hosmer and Lemeshow statistic to sample size, where small differences between estimated and observed risks can still produce a significant p-value in large sample size [11,26]. In general, low Brier scores were observed in the current study, lower compared to those reported by Masconi, Matsha [22]. However, a lower Brier score does not necessarily imply higher calibration [31].

The pattern of misprediction revealed that the Kuwaiti and the Cambridge models underestimated the probability of the low-risk group and overestimated the probability of the high-risk group, while the Omani model produced sufficient attenuation for a satisfactory calibration based on visual inspection. A good model would be one that would be able to i. identify those at high risk who need confirmation testing, ii. identify those at low risk to be screened out since the confirmation test is invasive and comes at additional costs. Thus, the misclassification as seen with the Kuwaiti and the Cambridge models would not affect their use for first-line screening of diabetes, more so when a threshold is set for absolute risk requiring a further confirmation test (i.e. ≥ 32 points for Kuwaiti and >0.37 for Cambridge). Therefore, an overestimation beyond such values does not cause a change in the public health strategy [26]. The other two models (Rotterdam and SUNSET) underestimated the probability of the high-risk group and overestimated that of the low-risk group and therefore could not be desirable for the screening of diabetes among such a population of African ancestry. Hence, the usage of the Rotterdam and SUNSET models would tend to subject people with low risk to a confirmation test and delay the diagnosis of those who have diabetes.

### 4.1 Strengths and limitations

The use of a large randomly selected homogeneous sub-Saharan African adult population living in Ghana and Europe with a wide age distribution and capturing a population at high risk through a random selection method limits potential selection bias a strength of this study. The finding of this study could therefore be potentially extrapolated to other sub-Saharan African ancestry migrants living in the western Europe and their counterparts living in West Africa. However, the extension of the current findings among people with African ancestry born in and living in high-income countries such as Europe and North America is uncertain, since the population of this study does not include significant numbers of second generation migrants. Another strength is that HbA1c was measured in one laboratory for samples from all sites. However, the use of HbA1c for the definition of screen-detected diabetes and prediabetes in an African population where there is a high prevalence of hemoglobinopathies and other conditions that may shorten the life of erythrocytes is a potential limitation due to misclassification arising as a result of underestimation [32,33]. Also, since there is limited overlap between the various available biochemical tests for diabetes, positive diabetes cases could remain undetected when using such a one-test definition, the false-negative cases could be present, which would lead to underestimation of the prevalence of diabetes and calibration of the model. However, the recalibration of the models through intercept adjustment attenuates such an effect. Another limitation of this study is the fact that the FINDRISC model was originally designed for the detection of incident diabetes prediction and therefore its estimate does not reflect the likelihood of being a prevalent undiagnosed diabetes case. However, the FINDRISC model has been used over the years as both a prevalent and an incidence model, and therefore adding it to the current analysis is appropriate [14,22,34].

## 5 Conclusion

Our findings suggest an acceptable discrimination ability of all six non-invasive risk screening models to detect diabetes and moderate discrimination to detect prediabetes among this population of African ancestry. Three out of the five models (Kuwaiti, Omani, and Cambridge) assessed for calibration were found to be useful in the estimation of the absolute risk after adjusting for the prevalence of screen-detected diabetes in the validation population. Further validation and recalibration of these models in non-migrant African and African migrant populations are needed before large-scale use is to be recommended.

## Authors’ contributions

JOY, APK, and CA conceived and designed the study. JOY and APK analysed the data. JOY, APK, and CA drafted the manuscript with the cooperation of all co-authors. All authors participated in the critical content review and the approval of the final manuscript.

## Funding

This work was supported by the 10.13039/501100000780European Commission under the Framework Programme (Grant Number: 278901). Professor Smeeth's contribution was supported by the 10.13039/100010269Wellcome Trust, grant number WT082178. Professor Karlijn A. C. Meeks is supported by the Intramural Research Program of the 10.13039/100000002National Institutes of Health in the 10.13039/100006090Center for Research on Genomics and Global Health (CRGGH). The CRGGH is supported by the 10.13039/100000051National Human Genome Research Institute, 10.13039/100000062National Institute of Diabetes and Digestive and Kidney Diseases, 10.13039/100000093Center for Information Technology, and 10.13039/100000179Office of the Director at the 10.13039/100000002National Institutes of Health (1ZIAHG200362).

## Availability of data

The datasets used for the current study are not publicly available data but can be accessed through a reasonable request to the RODAM advisory board.

## Declaration of competing interest

By letter the authors declare no competing interests. And that all funding sources for this work have been declared.
