# Supplementary material for: Validation of prevalent diabetes risk scores based on non-invasively measured predictors in Ghanaian migrant and non-migrant populations – The RODAM study
Source: Public Health Pract (Oxf). 2023 Nov 23;6:100453. doi: 10.1016/j.puhip.2023.100453 (PMC10687695; doi:10.1016/j.puhip.2023.100453)
Supplement: Multimedia component 1 [file mmc1.docx]

**Detailed Methods**

**Study population and design of the study**

In brief, the RODAM study is a multicentered population-based study involving a homogeneous sub-Saharan African adult population of Ghanaian ancestry living in Ghana and Europe. Between 2012 and 2015, using a cross-sectional approach, the study interviewed 6385 participants, physically examined, and attempted blood collection of 5898 adult Ghanaians in rural and urban Ghana, and in the cities of Amsterdam, Berlin, and London. Data collection was conducted by trained personnel using highly standardized tools and operating procedures including questionnaires/ interviews, physical examination, and biological samples ^14^. Details of captured variables and participants’ distribution by the site are published elsewhere ^14^.

**Criteria for inclusion in the current study**

As seen in Figure A1, the current analysis includes 4843 participants aged 25 - 70 years, who have no previous diagnosis of diabetes and were not on treatment for diabetes. Participants with a previous diagnosis of diabetes were defined as a positive answer to any of the questions, i. have you ever been diagnosed with diabetes by a doctor or health care worker? ii. has a doctor or specialist treated you for diabetes in the past 12 months? iii. do you use tablets for your diabetes? iv. are you on a diabetes diet right now? v. do you use insulin injections for your diabetes as well as diabetes treatment medication recorded in the participants' medication list at the registration desk on-site. Participants with no physical examination or incomplete variables were also excluded from the present analyses. (Figure 1A

**Physical Measurement**

Participants’ weight was measured twice to the nearest 0.1 kg with the SECA 877 scale, in light clothes, without shoes, and in a standing position with feet at an angle of 35° in the center of the scales. Arms were hanging loosely at their sides and heads facing forward. Using a portable stadiometer (SECA 217), the height of participants was measured to the nearest 0.1 cm. The waist circumference was measured twice at the nearest 0.1cm using a measuring tape at the point midway between the iliac crest and the costal margin (lower rib). The Body mass index (BMI) was derived by dividing the weight in kg by the square of height in meters(m^2^).

Blood pressure (BP) was measured three times using a validated semi-automated device (Microlife Watch BP home, Widnau, Switzerland), with appropriately sized cuffs after at least 5 min rest while seated. The mean of the last two BP measurements was used for the analyses. Hypertension was defined as systolic BP ≥ 140 mmHg and/or diastolic BP ≥ 90 mmHg, and/or being on antihypertensive medication treatment.

**Outcome Variable**

The outcome variable for the analysis was based on haemoglobin A1_C_ (HbA1c) levels. Whole blood was collected into an EDTA- vacutainer tube labelled with a barcode and temporarily stored on-site at -20 ^0^C and later at -80 ^0^C before being transported to the central laboratory at Charité-University Medicine Berlin (Berlin, Germany). The Tosoh G8 Glycohemoglobin Analyzer was used to measure HBA1c. The analyzer uses a non-porous ion-exchange high-performance liquid chromatography (HPLC) for the separation of haemoglobin fractions, which is measured photometrically at 415 nm. Screen-detected diabetes was defined as HbA1c ≥48 mmol/mol(6.5%) , prediabetes HbA1_C_ ≥39(5.7%) and < 48mmol/mol(6.5%) and normoglycemia HbA1_C_ <39 mmol/mol(5.7%). The main outcome variable was screen-detected diabetes, assessed against nondiabetes (HbA1_C_ < 48mmol/mol). Model ability to discriminate prediabetes from normoglycemia was also assessed (i.e. screen-detected diabetes was excluded from this subset of data). In a supplementary analysis, model performance in detecting hyperglycemia (HbA1_C_ ≥39mmol/moL(5.7%) from normoglycemia (HbA1_C_ <39 mmol/moL(5.7%) was assessed. Also, analyses were repeated using fasting blood glucose levels to define the outcome variable based on the following definitions: Normal glucose <5.6 mmol/l, Prediabetes ≥ 5.6 and <7 mmol/l, Diabetes ≥ 7 mmol/l.

**Statistical Methods**

The original regression equation coefficients with intercepts of the selected models were used for the computation of predictive risk scores for each participant. The variables used in the development of the models are shown in table S1 and the internal validation and external validation of models in the sub-Saharan African ancestry population are shown in table S2.

**Predictive Performance Measures**

The performance of the model was evaluated in terms of discrimination and calibration. Model discrimination was assessed using the area under the receiver operating characteristic curve (AUC) ^20^. AUC values range from 0.5 (no discrimination) to 1.0 (perfect discrimination) ^20^. AUC values ≥ 0.70 are generally classified as acceptable or moderate and values ≥ 0.80 are considered to be excellent ^21^. AUC comparison across models used non-parametric methods ^22^. The Youden index method was used to derive the optimal threshold point for each model, and the sensitivity and specificity of the models were estimated at this threshold ^23, 24^. MedCalc version 12.3.2 for Windows was used for data analysis.

Both graphical assessment and statistical test of goodness of fit, the Hosmer and Lemeshow statistic, where an insignificant test result indicates a good model fit, were used ^23, 25^ for calibration assessment. The population at risk was divided into deciles of ranked predicted risk. A bar graph of the decile of predicted risk and the observed event rate for each decile was plotted for the original model and after recalibration. Calibration graphs were plotted using the R package (RMS) version 4.1.0. The package also reported performance statistics. These statistics include i. the ordinal association between the observed and the expected (Sommers’ rank correlation) ranging from -1, when all pairs disagree, and 1, when all pairs agree, ii. the model intercept or calibration-in-the-large with a value of 0 for a perfect prediction, iii. the slope, which is a parameter of the regression model with the risk score as covariable serves as the shrinkage factor to account for overfitting ^26^; and iv. the Brier score (B), which is the mean squared error between the actual outcome and the estimated probabilities, ranging from 0 for a perfect prediction model to 0.25 for a noninformative model ^27^. This assessment was not done for the FINDRISC model, since the model was originally developed for estimating 10 years’ incidence of diabetes, thus the estimates do not represent the likelihood of being a prevalent undiagnosed case and therefore making a calibration analysis not useful.

**Recalibration**

Calibration is strongly affected by the outcome difference in the development and validation populations; a recalibration of the selected models was carried out through intercept adjustment ^5^. Using a correction factor based on the mean predicted risk and the prevalence in the validation set ^20^, the original models were updated by the addition of the correction factor to the intercept of the original model.

$$Correction factor=\ln\left( \frac{\frac{prevalence in the validation set}{1-prevalence in the validation set}}{\frac{mean predicted risk in the validation set}{1-mean predicted risk in the validation set}} \right)$$

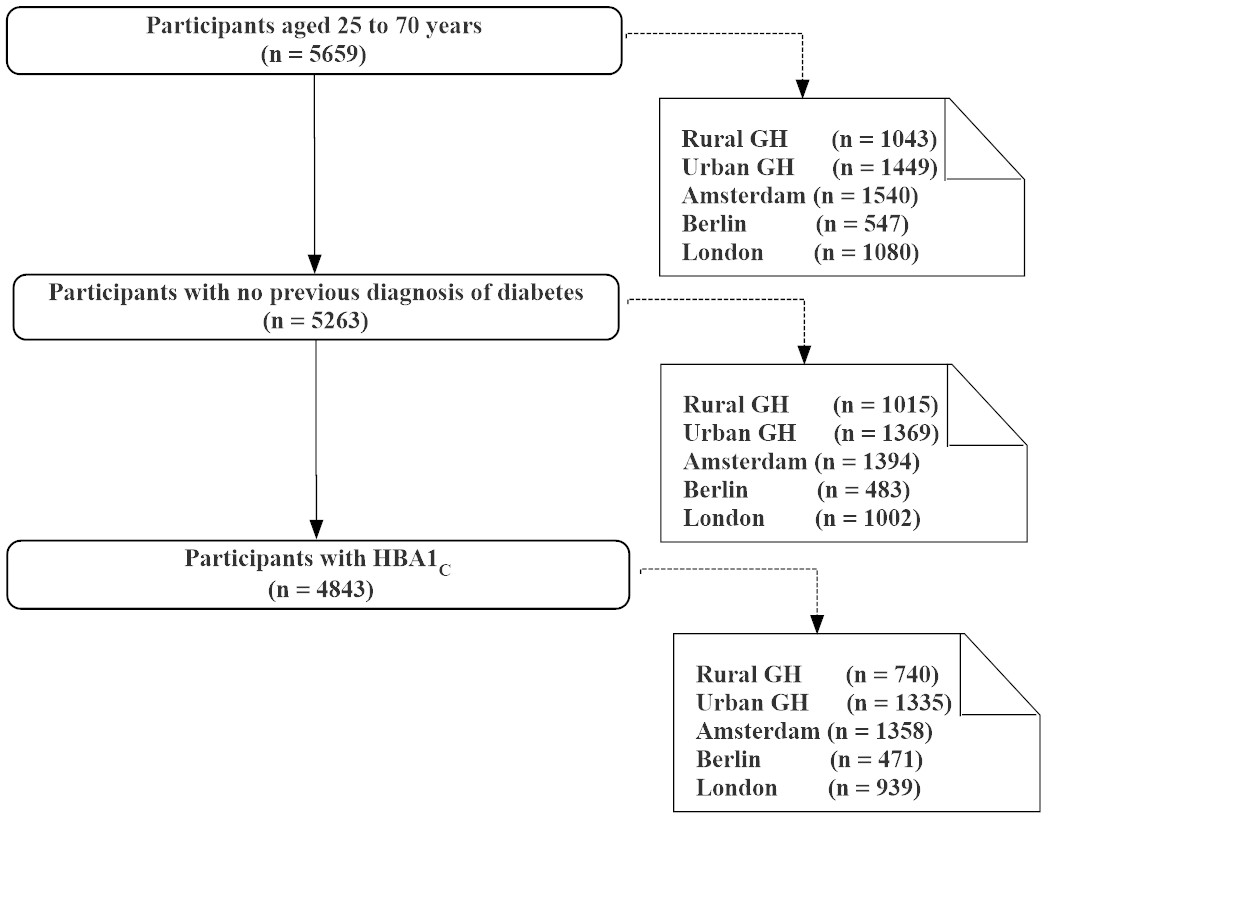


Figure A1: Flowchart of participants from the RODAM study included in the current analysis. GH, Ghana

Figure A2 : Comparative ROC Curves indicating performance of various diabetes predictive model in the prediction of screen-detected diabetes

Figure A3: Calibration plots for the prediction of screen-detected diabetes before and after recalibration through intercept adjustment: Dxy = Somers, rank correlation, R2 = R-square correlation, intercept, slope = shrinkage factor to account for overfitting, B=Brier score

Figure A4: Calibration plots for the prediction of screen-detected diabetes before and after recalibration through intercept adjustment: Dxy = Somers, rank correlation, R2 = R-square correlation intercept, slope = shrinkage factor to account for overfitting, B=Brier score

Figure A5: : Comparative ROC Curves indicating the performance of various diabetes predictive model in the prediction of prediabetes


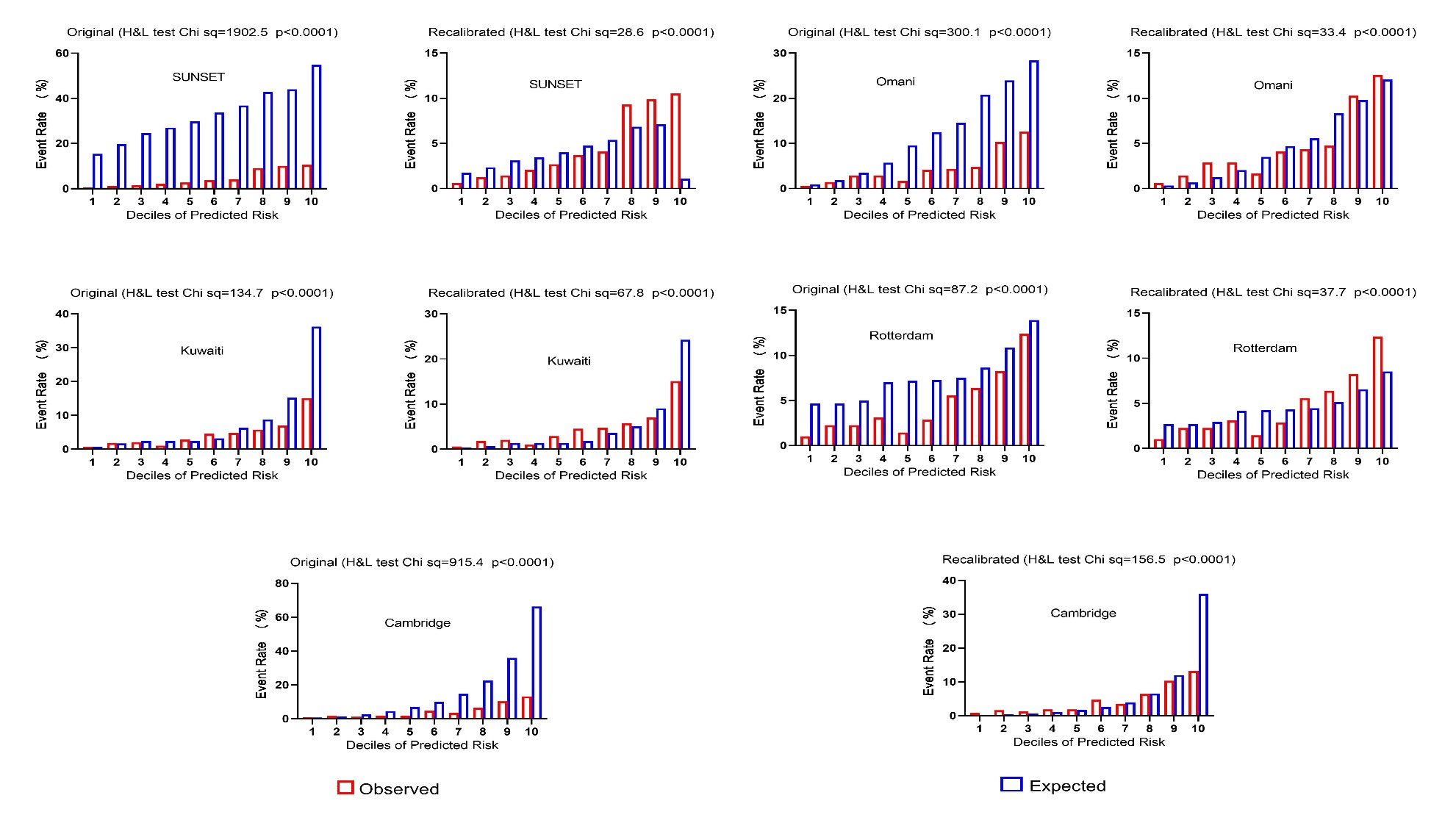


Figure A6: Plots of predicted versus observed diabetes prevalence rates across increasing deciles of predicted risk from models before and after recalibration through intercept adjustment. H&L -Hosmer & Lemeshow test p is significant ≥0.05.


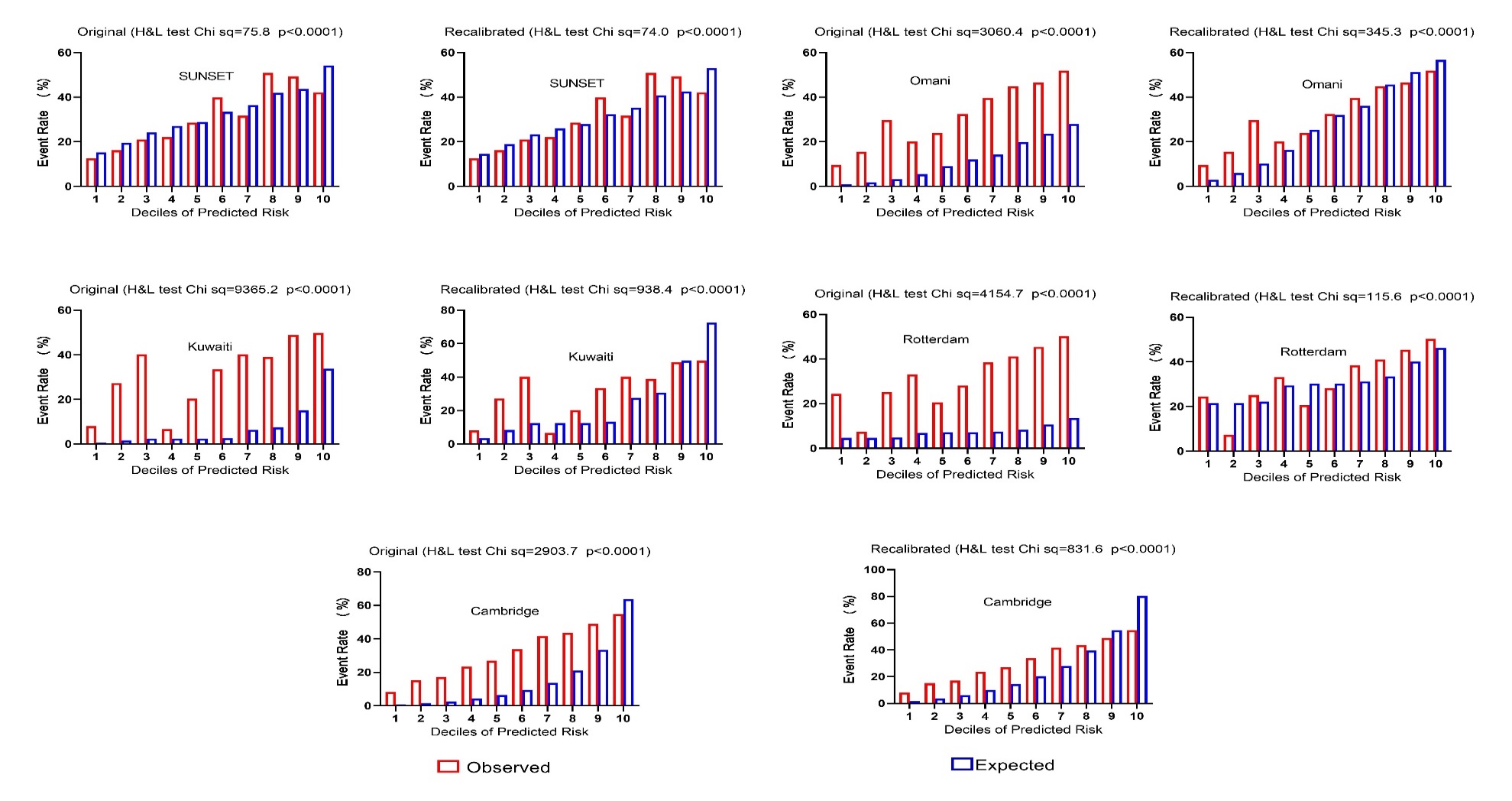


Figure A7: Calibration plots of diabetes models for the prediction of prediabetes among all participants before and after recalibration through intercept adjustment. H&L -Hosmer & Lemeshow test p is significant ≥0.05

**Table A1: Variables used in the development of diabetes risk model**

| Parameter | Cambridge | FINDRISC | Kuwaiti | Omani | Rotterdam | SUNSET |
| --- | --- | --- | --- | --- | --- | --- |
| Age | Y | Y | Y | Y | Y | Y |
| Sex | Y | Y | - | - | Y | - |
| Body mass index | Y | Y | - | Y | Y | Y |
| Waist circumference | - | Y | Y | Y | - | Y |
| Ethnicity | - | - | - | - | - | Y |
| Family history of diabetes | Y | Y | Y | Y | - | Y |
| Smoking status | Y | - | - | - | - | - |
| Hypertension | - | - | - | Y | - | Y |
| Antihypertensive medication | Y | Y | Y | - | Y | Y |
| Current steroid treatment | Y | - | - | - | - | - |
| Resting heart rate | - | - | - | - | - | Y |
| Family history of CVD | - | - | - | - | - | Y |
| Physical activity levels | - | Y | - | - | - | - |
| Fruit and vegetable intake | - | Y | - | - | - | - |

Y= variable found in the model

**Table A2: External validation of diabetes equations in an African Population**

| **Parameter** | **Developed population and internal validation** | **Results of external validation in African ancestry population** | **Population & Reference for external validation studies** |
| --- | --- | --- | --- |
| Cambridge | UK (1077); AUC: 0.80 | C-statistic: 0.67, Moderate | African South African; [23] |
|  |  | AUC: 0.67, Moderate | African Caribbean, [28] |
| FINDRISC | Finns (4435); AUC: 0.86 | AUC: 0.63; Moderate | African Botswana, [30] |
|  |  | AUC: 0.76; Acceptable | African American; [29] |
| Kuwaiti | Kuwaiti (562); AUC: 0.82 | C-statistic: 0.68; Moderate | African South African, [23] |
|  |  | AUC: 0.78; Acceptable | Ghanaians, [33] |
|  |  | AUC: 0.76; Acceptable | African Surinamese; [33] |
| Omani | Omani (4881) AUC: 0.83 | C-statistic: 0.66; Moderate | African South African, [23] |
|  |  | AUC: 0.74; Acceptable | Ghanaians, [33] |
|  |  | AUC: 0.77; Acceptable | African Surinamese, [33] |
| Rotterdam | Netherlands (1016); AUC: 0.74 | C-statistic: 0.64; Moderate | African South African, [23] |
|  |  | AUC: 0.75; Acceptable | Ghanaians; [33] |
|  |  | AUC: 0.81 | African Surinamese, [33] |
| SUNSET | Netherlands:  African Surinamese (593), AUC: 0.80  Hindustani Surinamese (336) AUC: 0.74  Ethnic Dutch (486); AUC: 0.78 | None | None |

AUC: Area under the receiver operating characteristic curve

**Table A3: Predictive performance of diabetes risk scores for prediabetes among the population of Ghanaians living in Europe and Ghana.**

| **Parameter** | **Cambridge** | **FINDRISC** | **Kuwaiti** | **Omani** | **Rotterdam** | **SUNSET** |
| --- | --- | --- | --- | --- | --- | --- |
| **All Individuals** | | | | | | |
| **AUC** | 0.70(0.69-0.71) | 0.66(0.64-0.67) | 0.66(0.65-0.67) | 0.68(0.66-0.69) | 0.64(0.63-0.66) | 0.67(0.65-068) |
| **Sensitivity** | 67.1% | 63.4% | 56.9% | 66.9% | 57.2% | 69.3% |
| **Specificity** | 64.2% | 62.6% | 67.6% | 61.4% | 66.7% | 58.1% |
| **Criterion** | >0.0896 | >0.036 | >0.0249 | >0.1171 | >0.0718 | >0.3243 |
| **p value** | <0.0001 | <0.0001 | <0.0001 | <0.0001 | <0.0001 | <0.0001 |
| **Male Ghanaians** | | | | | | |
| **AUC** | 0.70(0.68-0.72) | 0.65(0.63-0.67) | 0.67(0.65-0.69) | 0.69(0.66-0.71) | 0.66(0.63-0.68) | 0.68(0.66-0.70) |
| **Sensitivity** | 68.8% | 66.3% | 59.6% | 59.2% | 61.5% | 69.4% |
| **Specificity** | 62.4% | 56.8% | 67.2% | 69.3% | 65.1% | 58.2% |
| **Criterion** | >0.0914 | >0.010 | >0.0249 | >0.1347 | >0.0718 | >0.3243 |
| **p value** | <0.0001 | <0.0001 | <0.0001 | <0.0001 | <0.0001 | <0.0001 |
| **Female Ghanaians** | | | | | | |
| **AUC** | 0.70(0.68-0.72) | 0.68(0.66-0.69) | 0.65(0.64-0.67) | 0.67(0.66-0.69) | 0.63(0.61-0.65) | 0.66(0.64-0.68) |
| **Sensitivity** | 73.2% | 77.5% | 54.2% | 67.3% | 54.3% | 68.9% |
| **Specificity** | 58.9% | 53.8% | 68.9% | 60.9% | 67.6% | 58.3% |
| **Criterion** | >0.069 | >0.036 | >0.0447 | >0.1171 | >0.0718 | >0.3243 |
| **p value** | <0.0001 | <0.0001 | <0.0001 | <0.0001 | <0.0001 | <0.0001 |
| **Ghanaians Living in Ghana** | | | | | | |
| **AUC** | 0.71(0.69-0.73) | 0.68(0.66-0.70) | 0.68(0.66-0.70) | 0.69(0.67-0.71) | 0.64(0.62-0.66) | 0.68(0.66-0.70) |
| **Sensitivity** | 63.7% | 62.7% | 55.7% | 66.8% | 54.4% | 64.5% |
| **Specificity** | 70.8% | 68.6% | 72.2% | 67.4% | 70.0% | 64.1% |
| **Criterion** | >0.0882 | >0.035 | >0.0249 | >0.1171 | >0.0718% | >0.3243 |
| **p value** | <0.0001 | <0.0001 | <0.0001 | <0.0001 | <0.0001 | <0.0001 |
| **Ghanaians Living in Europe** | | | | | | |
| **AUC** | 0.66(0.65-0.68) | 0.62(0.60-0.64) | 0.64(0.62-0.65) | 0.65(0.63-0.67) | 0.62(0.61-0.64) | 0.64(0.62-0.65) |
| **Sensitivity** | 66.8% | 57.9% | 56.0% | 59.5% | 58.2% | 71.0% |
| **Specificity** | 58.4% | 61.4% | 63.8% | 62.8% | 62.9% | 51.9% |
| **Criterion** | >0.0943 | >0.041 | >0.0447 | >0.1213 | >0.0718 | >0.3243 |
| **p value** | <0.0001 | <0.0001 | <0.0001 | <0.0001 | <0.0001 | <0.0001 |

Data is presented as AUC (95% CI of AUC), AUC- Area under the curve. A score of 0.50 indicates no discrimination; 0.50<AUC<0.70 poor discrimination; 0.70≤AUC<0.80 acceptable discrimination; 0.80≤AUC<0.90 excellent discrimination; 0.90≤AUC outstanding discrimination; and a score of 1.00 perfect discrimination. Criterion is the threshold or cut-off score at which the discrimination is determined.

**Table A4:** **Predictive validity of diabetes risk scores for screen-detected diabetes among the population of Ghanaians living in Europe and Ghanaian living in Ghana stratified by gender.**

| **Parameter** | | **Cambridge** | **FINDRISC** | **Kuwaiti** | **Omani** | **Rotterdam** | **SUNSET** |
| --- | --- | --- | --- | --- | --- | --- | --- |
| **Ghanaians living in Europe** | | | | | |  |  |
| **Male** | **AUC** | 0.73(0.71-0.76) | 0.73(0.70-0.75) | 0.72(0.69-0.74) | 0.74(0.71-0.76) | 0.72(0.69-0.74) | 0.72(0.69-0.74) |
|  | **Sensitivity** | 67.53 | 61.04 | 58.44 | 64.94 | 76.62 | 72.73 |
|  | **Specificity** | 72.54 | 74.91 | 80.35 | 74.47 | 57.46 | 66.05 |
|  | **Criterion** | >0.2823 | >0.05 | >0.1082 | >0.1638 | >0.0718 | >0.3385 |
|  | **p value** | <0.0001 | <0.0001 | <0.0001 | <0.0001 | <0.0001 | <0.0001 |
| **Female** | **AUC** | 0.74(0.71-0.75) | 0.73(.70-0.75) | 0.74(0.70-0.75) | 0.71(0.67-0.72) | 0.71(0.68-0.72) | 0.7.0(0.66-0.71) |
|  | **Sensitivity** | 77.63 | 73.68 | 68.42 | 60.53 | 82.89 | 77.63 |
|  | **Specificity** | 60.37 | 68.22 | 69.08 | 76.07 | 50.67 | 56.63 |
|  | **Criterion** | >0.1479 | >0.0955 | >0.0636 | >0.2227 | >0.0691 | >0.3778 |
|  | **p value** | <0.0001 | <0.0001 | <0.0001 | <0.0001 | <0.0001 | <0.0001 |
| **Ghanaians Living in Ghana** | | | |  |  |  |  |
| **Male** | **AUC** | 0.79(0.76-0.82) | 0.77(0.74-0.81) | 0.74(0.72-0.79) | 0.80(0.78-0.84) | 0.67(0.64-0.71) | 0.83(0.81-0.87) |
|  | **Sensitivity** | 73.68 | 73.68 | 57.89 | 84.21 | 68.42 | 68.42 |
|  | **Specificity** | 77.03 | 70.27 | 79.88 | 69.82 | 66.82 | 83.33 |
|  | **Criterion** | >0.1188 | >0.018 | >0.0436 | >0.0863 | >0.0718 | >0.3243 |
|  | **p value** | <0.0001 | <0.0001 | <0.0001 | <0.0001 | 0.0037 | <0.0001 |
| **Female** | **AUC** | 0.71(0.69-0.74) | 0.72(0.70-0.75) | 0.71(0.69-0.73) | 0.68(0.66-0.69) | 0.66(0.64-0.69) | 0.74(0.72-0.77) |
|  | **Sensitivity** | 73.08 | 55.77 | 69.23 | 65.38 | 65.38 | 63.46 |
|  | **Specificity** | 66.06 | 79.13 | 64.62 | 65.7 | 61.66 | 77.91 |
|  | **Criterion** | >0.0839 | >0.09 | >0.0249 | >0.1213 | >0.0666 | >0.4199 |
|  | **p value** | <0.0001 | <0.0001 | <0.0001 | <0.0001 | 0.0001 | <0.0001 |

Data is presented as AUC (95% CI of AUC), AUC- Area under the curve. A score of 0.50 indicates no discrimination; 0.50<AUC<0.70 poor discrimination; 0.70≤AUC<0.80 acceptable discrimination; 0.80≤AUC<0.90 excellent discrimination; 0.90≤AUC outstanding discrimination; and a score of 1.00 perfect discrimination. Criterion is the threshold or cut-off score at which the discrimination is determined.

**Table A5: Predictive validity of screen-detected diabetes by diabetes risk scores among the population of Ghanaians stratified by gender and urbanization level.**

| **Parameter** | **Cambridge** | **FINDRISC** | **Kuwaiti** | **Omani** | **Rotterdam** | **SUNSET** |
| --- | --- | --- | --- | --- | --- | --- |
| **Population living in urban Ghana** | | | | | | |
| **AUC** | 0.71(0.68-0.73) | 0.71(0.69-0.74) | 0.70(0.67-0.72) | 0.69(0.67-0.72) | 0.64(0.62-0.67) | 0.75(0.72-0.77) |
| **Sensitivity** | 73.33 | 53.33 | 70 | 75 | 61.67 | 65 |
| **Specificity** | 63.23 | 81.65 | 63.31 | 57.14 | 64.01 | 77.36 |
| **Criterion** | >0.0946 | >0.0914 | >0.0249 | >0.1171 | >0.0718 | >0.3906 |
| **p value** | <0.0001 | <0.0001 | <0.0001 | <0.0001 | 0.0003 | <0.0001 |
| **Population living in rural Ghana** | | | | | | |
| **AUC** | 0.71(0.68-0.74) | 0.73(0.71-0.77) | 0.73(0.69-0.76) | 0.72(0.68-0.75) | 0.65(0.61-0.68) | 0.76(0.72-0.79) |
| **Sensitivity** | 45.45 | 72.73 | 45.45 | 63.64 | 63.64 | 63.64 |
| **Specificity** | 96.23 | 65.58 | 80.13 | 78.18 | 75.71 | 75.45 |
| **Criterion** | >0.2846 | >0.0242 | >0.0436 | >0.1213 | >0.0752 | >0.3243 |
| **p value** | 0.0189 | 0.0001 | 0.0047 | 0.0073 | 0.1145 | 0.0031 |
| **Male residents in urban Ghana** | | | | | | |
| **AUC** | 0.74(0.69-0.78) | 0.75(0.71-0.80) | 0.74(0.70-0.79) | 0.77(0.73-0.81) | 0.66(0.62-0.71) | 0.82(0.78-0.86) |
| **Sensitivity** | 73.33 | 53.33 | 66.67 | 86.67 | 66.67 | 66.67 |
| **Specificity** | 71.35 | 90.27 | 75.41 | 64.05 | 68.11 | 86.22 |
| **Criterion** | >0.117 | >0.0612 | >0.0436 | >0.0863 | >0.0718 | >0.3509 |
| **p value** | 0.0002 | 0.0008 | <0.0001 | <0.0001 | 0.0157 | <0.0001 |
| **Female residents in urban Ghana** | | | | | | |
| **AUC** | 0.70(0.67-0.73) | 0.70(0.67-0.73) | 0.69(0.66-0.72) | 0.67(0.64-0.70) | 0.64(0.61-0.67) | 0.71(0.68-0.74) |
| **Sensitivity** | 75.56 | 57.78 | 71.11 | 66.67 | 66.67 | 66.67 |
| **Specificity** | 59.6 | 76.62 | 58.62 | 61.8 | 56.31 | 72.89 |
| **Criterion** | >0.0873 | >0.0914 | >0.0249 | >0.1213 | >0.0666 | >0 .4199 |
| **p value** | <0.0001 | <0.0001 | <0.0001 | <0.0001 | 0.0009 | <0.0001 |
| **Male residents in rural Ghana** | | | | | | |
| **AUC** | 0.87(0.82-0.90) | 0.82(0.77-0.87) | 0.65(0.64-0.75) | 0.83(0.78-0.87) | 0.69(0.63-0.75) | 0.77(0.74-0.84) |
| **Sensitivity** | 75 | 100 | 25 | 75 | 75 | 75 |
| **Specificity** | 97.97 | 66.55 | 99.66 | 86.15 | 74.32 | 69.93 |
| **Criterion** | >0.2805 | >0.0097 | >0.0636 | >0.1171 | >0.0855 | >0.2374 |
| **p value** | 0.0002 | <0.0001 | 0.2105 | 0.0046 | 0.1033 | 0.028 |
| **Female residents in rural Ghana** | | | | | | |
| **AUC** | 0.66(0.63-0.72) | 0.71(0.66-0.75) | 0.74(0.70-0.78) | 0.64(0.61-0.70) | 0.67(0.62-0.71) | 0.74(0.70-0.78) |
| **Sensitivity** | 57.14 | 57.14 | 57.14 | 57.14 | 57.14 | 71.43 |
| **Specificity** | 80.38 | 82.70 | 76.79 | 73.21 | 82.28 | 71.52 |
| **Criterion** | >0.0835 | >0.0708 | >0.0436 | >0.1213 | >0.0752 | >0.3385 |
| **p value** | 0.1742 | 0.1082 | 0.0122 | 0.2132 | 0.2608 | 0.0273 |

Data is presented as AUC (95% CI of AUC), AUC- Area under the curve. A score of 0.50 indicates no discrimination; 0.50<AUC<0.70 poor discrimination; 0.70≤AUC<0.80 acceptable discrimination; 0.80≤AUC<0.90 excellent discrimination; 0.90≤AUC outstanding discrimination; and a score of 1.00 perfect discrimination. Criterion is the threshold or cut-off score at which the discrimination is determined.

**Table A6: Predictive validity of screen-detect diabetes and prediabetes by diabetes risk score among the population of Ghanaians using fasting blood glucose**

| **Parameter** | **Cambridge** | **FINDRISC** | **Kuwaiti** | **Omani** | **Rotterdam** | **SUNSET** |
| --- | --- | --- | --- | --- | --- | --- |
| **All Individuals Diabetes** | | | | | | |
| **AUC** | 0.52(0.50-0.53) | 0.51(0.49-0.53) | 0.52(0.50-0.54) | 0.50(0.48-0.52) | 0.52(0.50-0.54) | 0.50(0.49-0.52) |
| **Sensitivity** | 54.4% | 55.4% | 29.4% | 20.7% | 77.2% | 32.6% |
| **Specificity** | 53.8% | 52.4% | 78.5% | 73.9% | 27.9% | 75.2% |
| **Criterion** | >0.0882 | >0.03 | >0.1081 | >0.0452 | >0.0465 | >0.2271 |
| **p value** | 0.6072 | 0.7252 | 0.5420 | 0.9792 | 0.5251 | 0.8984 |
| **All Individuals Prediabetes** | | | | | | |
| **AUC** | 0.54(0.52-0.55) | 0.54(0.52-0.55) | 0.53(0.51-0.54) | 0.52(0.50-0.53) | 0.53(0.51-0.54) | 0.54(0.52-0.56) |
| **Sensitivity** | 31.1% | 82.2% | 47.3% | 46.7% | 28.3% | 72.3% |
| **Specificity** | 76.6% | 23.8% | 59.7% | 59.9% | 78.3% | 35.8% |
| **Criterion** | >0.2167 | >0.0088 | >0.0249 | >0.1213 | >0.0855 | >0.3697 |
| **p value** | 0.0108 | 0.0129 | 0.06.9 | 0.2890 | 0.0671 | 0.0021 |

Data is presented as AUC (95% CI of AUC), AUC- Area under the curve. A score of 0.50 indicates no discrimination; 0.50<AUC<0.70 poor discrimination; 0.70≤AUC<0.80 acceptable discrimination; 0.80≤AUC<0.90 excellent discrimination; 0.90≤AUC outstanding discrimination; and a score of 1.00 perfect discrimination. Criterion is the threshold or cut-off score at which the discrimination is determined.
